# Effects of Applied Potential and Reactants to Hydrogen-Producing Biocathode in a Microbial Electrolysis Cell

**DOI:** 10.3389/fchem.2018.00318

**Published:** 2018-08-15

**Authors:** Swee Su Lim, Byung Hong Kim, Da Li, Yujie Feng, Wan Ramli Wan Daud, Keith Scott, Eileen Hao Yu

**Affiliations:** ^1^School of Engineering, Newcastle University, Newcastle Upon Tyne, United Kingdom; ^2^Fuel Cell Institute, Universiti Kebangsaan Malaysia, Bangi, Malaysia; ^3^Bioelectrochemistry Laboratory, Water Environment and Remediation Research Centre, Korea Institute of Science and Technology, Bongdong-eup, South Korea; ^4^State Key Laboratory of Urban Water Resource and Environment, School of Environment, Harbin Institute of Technology, Harbin, China

**Keywords:** hydrogen-producing biocathode, microbial electrolysis cell, electron bifurcation, sulfate reduction, bicarbonate conversion

## Abstract

Understanding the mechanism of electron transfer between the cathode and microorganisms in cathode biofilms in microbial electrolysis cells (MECs) for hydrogen production is important. In this study, biocathodes of MECs were successfully re-enriched and subjected to different operating parameters: applied potential, sulfate use and inorganic carbon consumption. It was hypothesized that biocathode catalytic activity would be affected by the applied potentials that initiate electron transfer. While inorganic carbon, in the form of bicarbonate, could be a main carbon source for biocathode growth, sulfate could be a terminal electron acceptor and thus reduced to elemental sulfurs. It was found that potentials more negative than −0.8 V (vs. standard hydrogen electrode) were required for hydrogen production by the biocathode. In additional, a maximum hydrogen production was observed at sulfate and bicarbonate concentrations of 288 and 610 mg/L respectively. Organic carbons were found in the cathode effluents, suggesting that microbial interactions probably happen between acetogens and sulfate reducing bacteria (SRB). The hydrogen-producing biocathode was sulfate-dependent and hydrogen production could be inhibited by excessive sulfate because more energy was directed to reduce sulfate (*E*° ${\text{SO}}_{4}^{2-}$/H_2_S = −0.35 V) than proton (*E*° H^+^/H_2_ = −0.41 V). This resulted in a restriction to the hydrogen production when sulfate concentration was high. Domestic wastewaters contain low amounts of organic compounds and sulfate would be a better medium to enrich and maintain a hydrogen-producing biocathode dominated by SRB. Besides the risks of limited mass transport and precipitation caused by low potential, methane contamination in the hydrogen-rich environment was inevitable in the biocathode after long term operation due to methanogenic activities.

## Introduction

Since hydrogen-producing biocathode was first introduced by Rozendal et al. (2008), biocathode activities in microbial electrolysis cells (MECs) were extensively studied. Combining wastewater treatment and production of hydrogen as energy carrier makes MECs an attractive technology. As the catalysts used in the cathode are living microorganisms, the associated microbiological knowledge is important for systematic optimisation MECs (Kim et al., 2015). Rozendal et al. (2008) used three phase start-up procedures to enrich hydrogen-producing biocathodes in a bioelectrochemical system (BES). A biocathode was obtained by reversing a bioanode. The whole process took less than a month to achieve a fully developed biocathode. Community analysis confirmed that sulfate-reducing bacteria (SRB) belonging to the genus, *Desulfovibrio*, were the key players in the hydrogen-producing biocathode (Croese et al., 2011, 2014). *Desulfovibrio sp*. conserve energy through a hydrogen cycling mechanism, that involves different types of hydrogenases which are involved in hydrogen production and consumption. A decade after, Jourdin et al. (2015) successfully grew an autotrophic biocathode and operated it for 9 months. They claimed that a sustainable autotrophic biocathode was involved in hydrogen evolution, when suitable cathodic condition were applied with inorganic carbon as the carbon source. The bacteria communities on the biocathode changed over the biofilm enrichment period; a significant increase on *proteobacteria* distribution between initial inoculum and enriched biocathode from 10 to 57% at the end of the experiment. Initial *Archaea* distribution disappeared completely from 30.3% to less than 0.1% of population. In additional to carbonates serving as the carbon source, both studies added a trace amount of sulfate into the catholyte to grow and maintain their biocathodes. SRB thrived and their domination could be due to the availability and quantity of sulfate present in the catholyte. It also been showed that sulfate was an important final electron terminal accepter in SRB hydrogen cycling mechanism (Kim and Gadd, 2008; Keller and Wall, 2011; Madigan et al., 2014). Nevertheless hydrogen production in a SRB dominated biocathode was the main purpose of the studies. Considering the standard reduction potentials of hydrogen and sulfate, hydrogen (*E*° H^+^/H_2_ = −0.41 V) requires more energy than sulfate reduction (*E*° ${\text{SO}}_{4}^{2-}$/H_2_S = −0.35 V). Furthermore, as the reduction potentials are relatively close (−0.06 V), indicates that sulfate reduction could take place in conjunction with hydrogen evolution, and the concentration of sulfate present may impact hydrogen production. Regardless of the standard reduction potential, many studies used much lower potential than −0.41 V in practical condition for biological hydrogen evolution (Geelhoed et al., 2010; Jeremiasse et al., 2012; Batlle-Vilanova et al., 2014; Jourdin et al., 2015). If SRB play an important role in electrochemical hydrogen production, sulfate concentration and its availability should be taken consideration as it will not only affect the current density of BES but also the working potential applied to the cathode. Bicarbonate (carbon source) and ammonium (nitrogen source) were commonly used in the biocathode study which have direct link to the growth of biocathode but not the case where sulfate is the responsible as electron acceptor and sulfur source. Therefore, sulfate could be the third important parameter after the carbon and nitrogen sources. Some studies presented results where additional acetate could enhance the start-up process of biocathode (Jeremiasse et al., 2012) or by using lactate as organic carbon with high sulfate concentration in pure culture tests (Aulenta et al., 2012). Due to the fact that SRB especially *Desulfovibrio* sp. cannot use inorganic carbon directly as a carbon source, there must be an active interaction between the species and other autotrophic bacteria in the hydrogen-producing biocathode to use the inorganic carbon as organic carbon. The community interaction between SRB and autotroph acetogens actually happened where only inorganic carbon, such as carbonates were in the solution (Muyzer and Stams, 2008; Mand et al., 2014). Even though SRB specifically *Desulfovibrio* sp. were found responsible for hydrogen production in BES biocathode, questions on optimum operational conditions and the feasibility of the biocathode in real applications still remain unanswered. The changes of influent content in varies inorganic carbon, nitrogen source and sulfate concentrations could shift microbial metabolism and the community and affect whole BES performance.

To fully understand the operational conditions of hydrogen-producing biocathode in a microbial electrolysis cell (MEC), the study of essential parameters and community interaction need to be integrated. Mand et al. (2014) proposed that sulfate-reducing bacteria and acetogen's interaction were responsible for steel pipe corrosion. However, other evidence showed that the form of ferrous sulfide layer on an iron sheet due to SRB corrosion was more severe without the sources of organic carbons or presence of acetogens (Venzlaff et al., 2013). The deposited ferrous sulfide works as a semiconductor in anaerobic corrosion by mediating electron flow from metal to the cells and by by-passing the slow reduction of proton to free hydrogen. The mechanisms of electron transfer are similar to a biocathode enriched from a mixed culture aimed for hydrogen production and could serve as a model for biocathode community interactions. Meanwhile Keller and Wall (2011) studied genetics and molecular level of electron flow in *Desulfovibrio* sp. for sulfate respiration. They reported how the respiration could assist in hydrogen production while reducing sulfate to sulfides. The results also inferred that periplasm hydrogenases plays an important role in hydrogen evolution. However, no experiment has been conducted to further examine the hypothesis. In addition, Geelhoed et al. (2010) discussed how the key enzymes, [Fe-Fe]- and [NiFe]-hydrogenases, from *Desulfovibrio vulgaris* were involved in hydrogen production. They stressed that utilization of immobilized whole cells were better and more robust than using only enzymes and therefore co-culture should be considered. As the whole cells and community should be focused, electron transfer within syntrophic partners become important and, from a thermodynamic point of view, hydrogen production via reduction of proton has to be coupled with energy conservation from hydrogenases. The balance between the conservation energy and hydrogen production indicated that microbial communities in a biocathode are able to grow and maintain their catalytic activity. It was also suggested that studying the correct growth conditions with a carbon source and applied voltage, longevity of the biocathode could be the key issues for further understanding the electron transport mechanism. Later, Rosenbaum et al. (2011) proposed possible direct and indirect electron transfer mechanisms by analyzing the literature on hydrogen producing biocathodes. On one hand, direct mechanisms were involved in direct electron transfer through *c*-type cytochromes either coupled with or without hydrogenases. On the other hand, indirect electron transfer mechanism relied on natural redox mediators shuttling between cathode and hydrogenases. Surprisingly, they suggested that the biocatalysed reactions was not necessarily an energy conservation process for microorganisms (Rosenbaum et al., 2011). Recently, Kim et al. (2015) proposed another electron transfer mechanism, similar to those in microbial influenced corrosion (MIC) and showed a sound reason that biocathode should conserve energy during electron consuming reactions, i.e., microbes performed proton reduction and should grow and be maintained under the given cathodic condition for sustainable function and thermodynamically balance.

The objective of the study was to re-culture biocathodes to optimize operational conditions and increase biocathode performance for hydrogen production, by manipulating the cathode potential, inorganic carbon and sulfate concentrations. The study will help to determine what kind of wastewaters will be suitable for biocathode formation and assist in establishing potential electron transfer mechanisms. It will also indicate the possible wastewater treatments that could be performed using this technology.

## Materials and methods

### Experimental setup and biocathode enrichment

**Figure 1:**
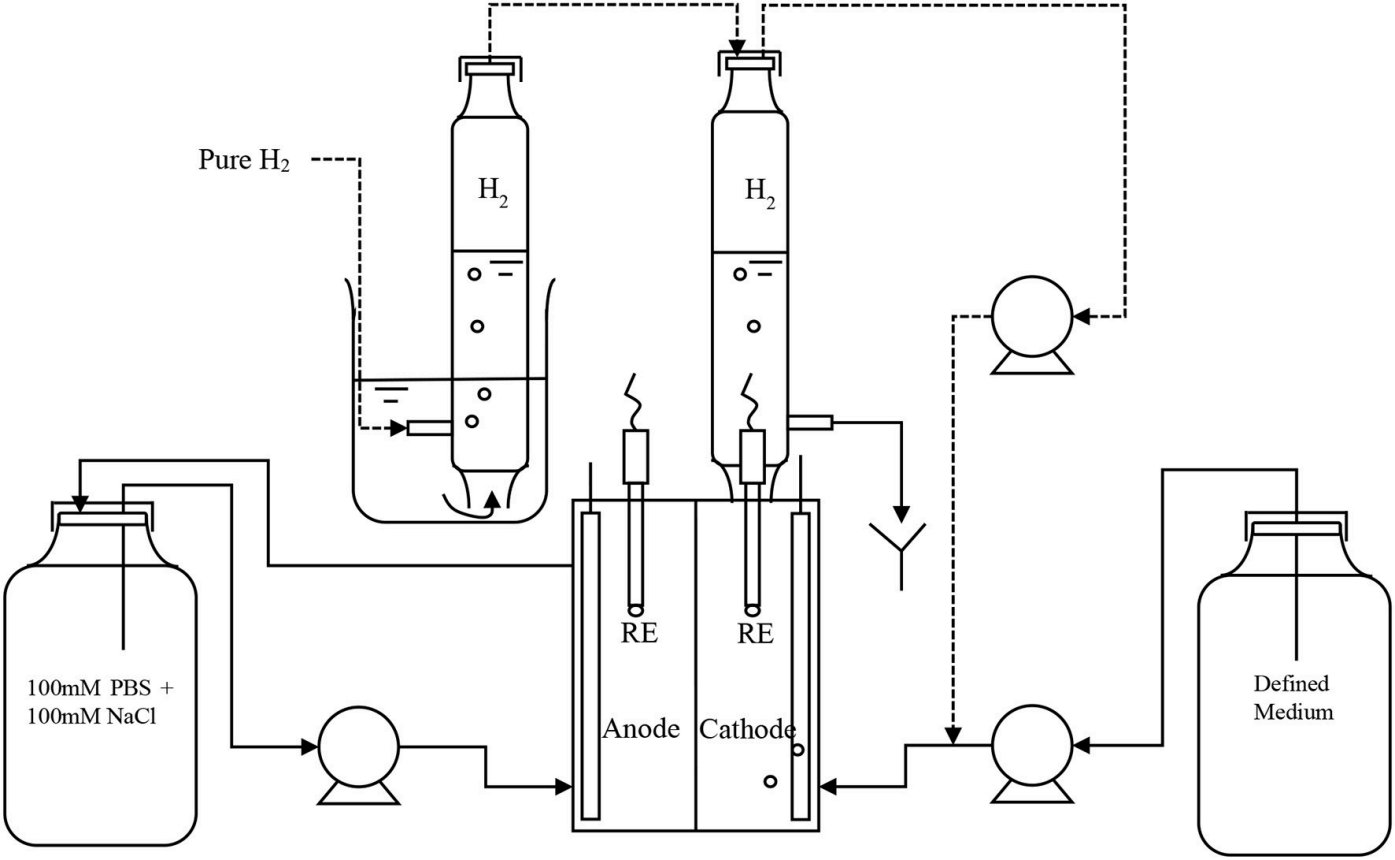
Schematic of the experimental setup.

Double-chamber electrochemical cells, 25 cm^3^ (mL) in volume (each chamber) were used as described in Lim et al. (2017). Figure 1 is the schematic of the experimental setup in this study. The enrichment of hydrogen-producing biocathode was performed as stated in Rozendal et al. (2008). A three step start-up procedure and polarity reversal method was exploited to obtain the desired biocathode. An abiotic anode (RVG-2000, Mersen, USA) coated with 0.5 mg/cm^2^ platinum catalyst was used. Anolyte was a mixture of sodium chloride and phosphate buffer consisted of (g/L): NaH_2_PO_4_·2H_2_O 3.30; Na_2_HPO_4_·2H_2_O 5.14; NaCl 2.92. The anolyte was circulated from a 250 mL reservoir to anodic chamber at flowrate 8.7 mL/min. Once a stable current was observed, the biocathode potential was further increased and fixed at −1.0 V versus standard hydrogen electrode (SHE) for all the experiments unless stated otherwise. The catholyte medium contained (g/L): NaH_2_PO_4_·2H_2_O 0.66; Na_2_HPO_4_·2H_2_O 1.03, KHCO_3_ 1.0, NH4Cl 0.27, MgSO_4_·7H_2_O 1.23, CaCl_2_·2H_2_O 0.01 and trace element mixture 1.0 mL/L (Rozendal et al., 2008). The medium consisted of only phosphate buffer was first prepared and autoclaved. The remaining ingredients were filter-added then after. The amount of KHCO_3_ and MgSO_4_·7H_2_O was added into the medium as stated above except if mentioned otherwise. The medium was then fed continuously into the cathodic chamber at 0.2 mL/min. The anolyte consisted 5 times higher concentration of phosphate buffer than in catholyte when the solutions were prepared. It is to ensure anolyte pH was maintained in neutral under recycle condition. Ion balance could affect conductivity value in the electrolytes and performance of MEC due to different phosphate buffer concentration. However, the effect was insignificant in our study as small operation volume (25 mL each chamber with half of the volume filled with carbon felt electrode) and a closer electrode gap (≤1.0 cm) was used. During the enrichment process, hydrogen was filled in cathode headspace and recycled by a peristaltic pump into the cathode chamber and then bubbled through the catholyte. The headspace hydrogen was refilled every day.

### Experimental parameter

Enriched biocathodes were subjected to three main experiments to examine optimum conditions for better performance especially in producing hydrogen. The experiments include manipulating applied potentials and various sulfate and bicarbonate concentrations to the cathodes. Table 1 shows the experiment parameters used in this study. The applied potential experiments were done using chronoamperometry to check the biocathode performance in term of hydrogen production and their energy requirement in term of current. All experiments were conducted in duplicate. The average values with the maximum and minimum are presented.

**Table 1:** Experimental matrix.

**Parameter 1**	**Parameter 2**	**Parameter 3**
**Cathodic potential (V vs. SHE)**	**[Sulfate] mg/L (mM)**	**[Bicarbonate] mg/L (mM)**
0.5	0 (0)	0 (0)
0.7	96 (1)	61 (1)
0.8	288 (3)	183 (3)
0.9	768 (8)	305 (5)
1.0	–	610 (10)
–	–	3051 (50)

### Electrochemical analysis

Cyclic voltammetry (CV) was carried out after each experiment to compile the information how the catalytic activity responses to the experimental parameters. Four channel potentiostat (Quad, Whistonbrook Technologies, UK) was used to conduct the analysis. Start and end potentials were 0 and 1.0 V with scan rate 0.001 V/s and repeated for at least 3 cycles to obtain a stable voltammogram. Only the last voltammograms from the last cycles of each experiment are reported in this study. All potential values were reported as vs. SHE unless stated otherwise.

### Samples analysis and calculations

Influent and effluent samples were collected for each parameter test. Ph and conductivity are the simpler indication of the change liquid properties through bioelectrochemical reactions. For instance, substrate oxidation or proton reduction in anode or cathode could result the decrease or increase of pH value. While ionic conductivity may influence the efficiency of whole system when the reactant and product contents vary in electrolytes. pH (HI 9025 Microcomputer pH meter, Hanna Instruments, UK) and conductivity (HI 8733 Conductivity meter, Hanna Instruments, UK) values were measured for each sample before the sample was filtered through a 0.2 μm PES membrane [VWR (514-0072), UK]. The filtered samples were then kept in refrigerator under 4°C prior analysis.

Sulfate and total soluble carbon were the two main parameter in this study. It is important to monitor the changes of the sample contents and the effect of applied cathode potential. Anions compounds included sulfate (${\text{SO}}_{4}^{2-}$) and phosphate (${\text{PO}}_{4}^{3-}$) were determined by ion chromatography (Interrion HPIC, Dionex, USA) equipped with autosampler (AS-AP, Dionex, USA) while inorganic and organic carbon measured by total carbon analyzer (TOC-5050A, Shimadzu, UK) equipped with autosampler (ASI-5000A, Shimadzu, UK). The pH of TOC samples were maintained as they were collected. The alkaline condition of the samples avoid dissolution of carbonates to CO_2_ which could affect the results of total carbon.

Ammonium ion could contribute to the ionic strength of the medium while acted as nitrogen source to bioanode. Therefore, it was included in the analysis apart from the main parameter analysis. Ammonium (NH4-N) contain was determined by using the cell test kits (14559: 4.0–80.0 mg/L NH4-N) supplied by Merck, UK. The samples were prepared and added into the reagent vials according to the manufacture's procedures and then measured by a spectrophotometer (Spectroquant® Pharo 300, Merck, UK).

Hydrogen is the main product in this study. In order to calculate the hydrogen production rate, gas evolution from the biocathode was measured using a water replacement method. A gas collection tube with marked volume was placed on the top of cathodic chamber and then filled with catholyte from the top opening. Gas bubble produced from cathodic was evolved to the top of the tube and replaced the catholyte by pushing it out from a side outlet. The effluent channel was filled with catholyte all the time to maintain anaerobic condition and atmospheric pressure inside the chamber (Lim et al., 2017). The gas samples then were analyzed using a gas chromatography (GC-8A, Shimadzu, UK). Two columns molecular sieve 5A (mesh range 40-60) and Chromosorb 101 (mesh range 80–100) were equipped and operated at isothermal temperature 40°C. The carrier gas was research grade 99.99% N_2_ (BOC, UK) at a pressure of 100 kPa. A thermal conductivity detector was used to detect the gas based on their retention times. The actual hydrogen volume was calculated as

(1) $$\begin{array}{r} {\text{V}}_{\text{H}2}={\text{V}}_{\text{h}}\cdot {\text{X}}_{\text{H}2} \end{array}$$

where V_H2_ (L) is pure hydrogen volume, V_h_ (L) is the headspace volume of the gas captured in the glass collection tube, X_H2_ is fraction of hydrogen in the gas samples determined from the GC analysis. The actual hydrogen volume was then used to determined hydrogen production rate as

(2) $$\begin{array}{r} {\text{Q}}_{\text{H}2}={\text{V}}_{\text{H}2}/\left({\text{A}}_{\text{cat}}\cdot \text{t}\right) \end{array}$$

where Q_H2_ (L H_2_/m^2^ cathode/day) is hydrogen production rate, A_cat_ (m^2^) is cathode surface area and t (day) is production time.

Faraday's law of electrolysis equation was obtained to compute hydrogen recovery efficiency from cathode

(3) $$\begin{array}{r} {\text{r}}_{\text{cat}}\left(\%\right)={\text{Q}}_{\text{recovery}}/{\text{Q}}_{\text{supply}} \end{array}$$

where Q_recovery_ (C) = η·F·z is charge use to reduce proton to hydrogen, η is hydrogen recovery in mole, F is faraday constant (96,485 C/mol), z is the valency number of hydrogen formation which is 2. Q_supply_ (C) = ∫ I (t) dt is total charge supplied from a power supply within specific time of recovery.

And, energy yield from hydrogen relatives to electrical input is calculated based on

(4) $$\begin{array}{r} \eta_{\text{e}}\left(\%\right)={\text{W}}_{\text{h}}/{\text{W}}_{\text{e}}\times 100\% \end{array}$$

where W_h_ and W_e_ (J) are energy content of H_2_ and electrical energy.

## Results and discussion

### Enrichment of hydrogen-producing biocathode

**Figure 2:**
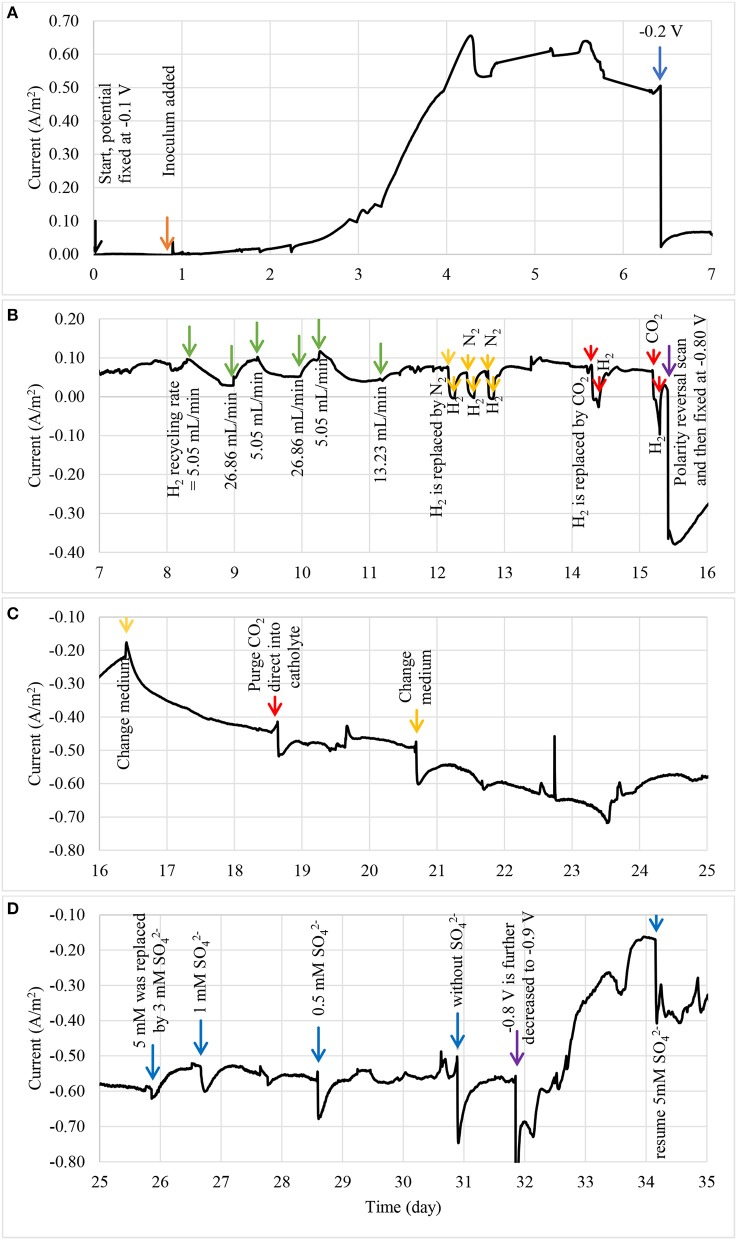
The current density profile of enriched bioelectrode using three step start-up procedure: **(A)** bioelectrode was enriched as bioanode between 0 and 7 day, and **(B)** subjected to series of bioanode confirmation tests between 7 and 16 day, **(C)** the bioanode was then switched to biocathode and grew under a fixed potential of −0.8 V between 16 and 25 day, and **(D)** subjected to sulphate tests after a stable current was observed between 25 and 35 day.

**Figure 3:**
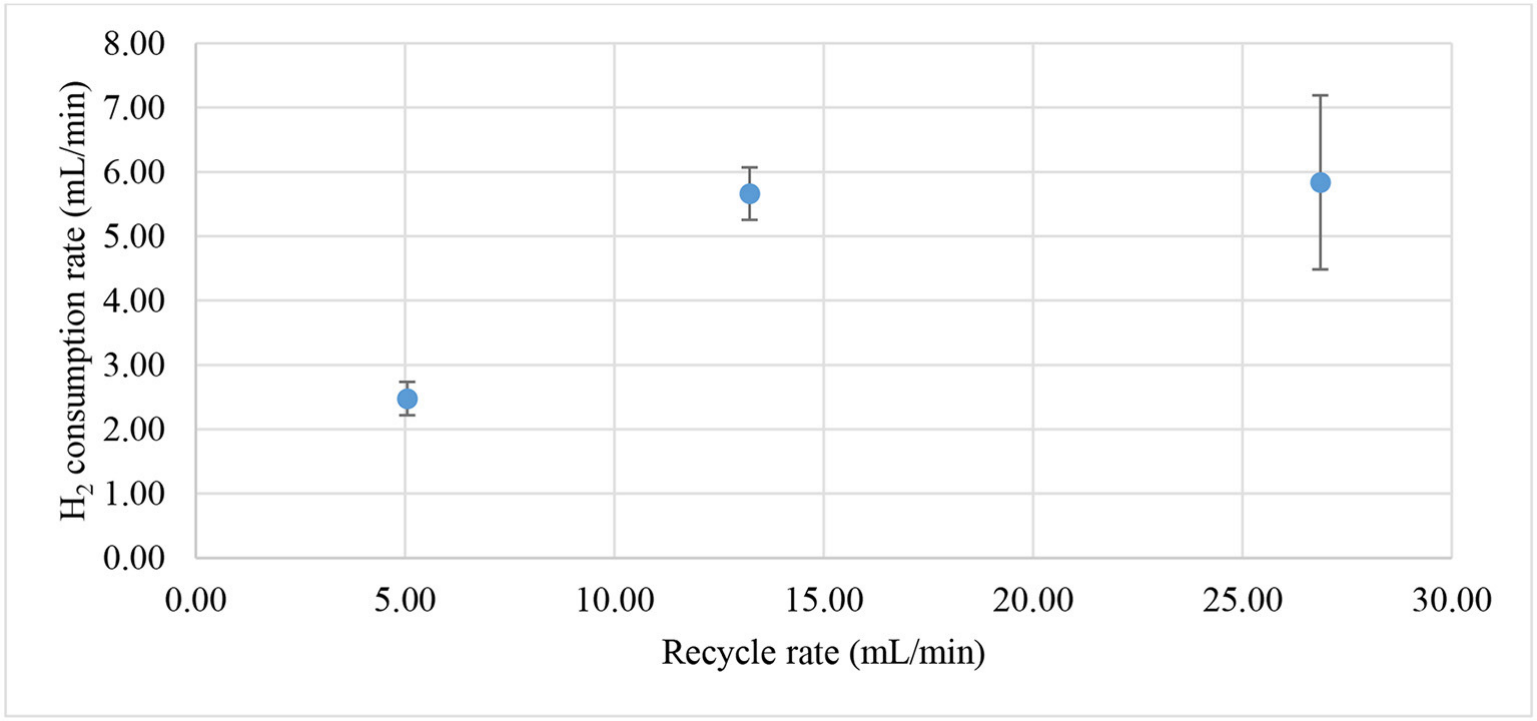
Hydrogen consumption rate based on hydrogen recycling rate from the headspace. Maximum hydrogen consumption was observed after the recycle rate was higher than 13 mL/min.

**Figure 4:**
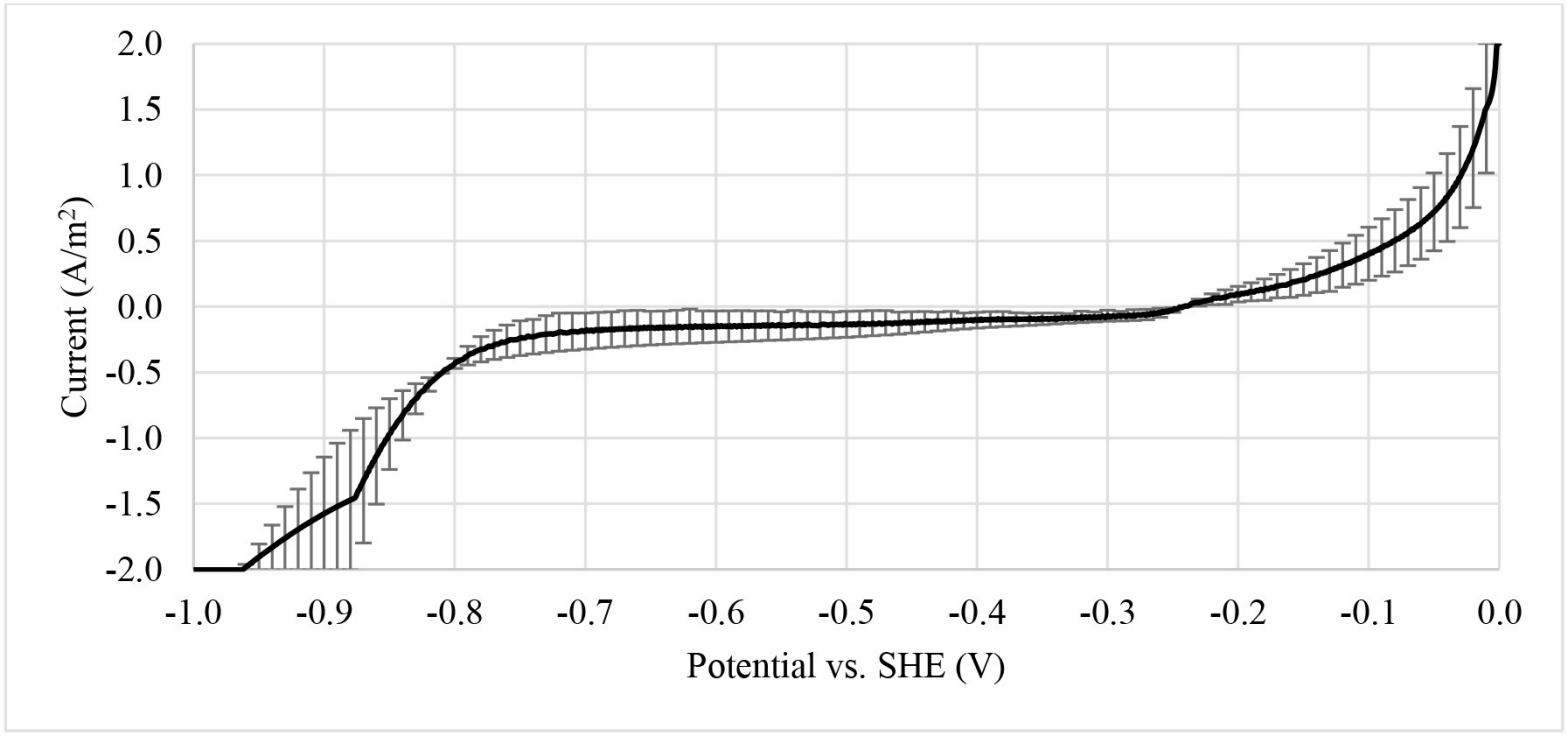
Polarity reversal scan from 0 to −1.0 V vs. SHE at scan rate of 0.2 mV/s. The information was obtained to determine the minimum potential to be fixed on bioelectrode for hydrogen production.

The enrichment of biocathodes in this study was performed by following the method reported in Rozendal et al. (2008). Cathode chamber was cultivated with inoculum collected from bioanode effluent operated in microbial fuel cell mode for over a year (Spurr, 2016). The inoculum was dominated by *Deltaproteobacteria* (~60%), followed by Clostridia (~20%) and *Bacteroidia* (~10%). The *Geobacter* sp. (~50%) was found as dominated ribotype in *Deltaproteobacteria* cluster and sulfate-reducing bacteria only consisted around 2%. A defined medium was prepared as described in Rozendal et al. (2008). Figure 2 shows the monitored current density during enrichment process. Target electrode was fixed at −0.1 V and was left for overnight without any inoculum. Acetate was used as electron donor to grow the bioelectrode. Significant current increase was observed after day 2 and a stable current was achieved after day 4. After 6.5 days, the potential was further reduced to −0.2 V and acetate was removed and replaced by hydrogen on the headspace. Hydrogen recycle rate was reduced and then increased in between 5.05 and 26.86 mL/min after 8 days of enrichment to check whether the bioelectrode was actively growth under hydrogen as electron donor. Figure 3 shows the relationship between hydrogen consumption and the rate of recycle between headspace and bioelectrodes. The optimum recycle rate was determined as 13 mL/min and was used throughout the rest of experiments. The biocathode test was continued by replacing hydrogen with nitrogen between 12 and 13 day. This is to confirm that the biocathode was relied and grew on hydrogen. After the test, carbon dioxide was filled instead of hydrogen. As the current value changed from positive to negative between 14 and 15.5 day, it justified that the polarity could be reversed from electron-producing bioanode to electron-accepting biocathode. A polarity reversal scan was performed at 15.5 day and the result is shown in Figure 4. Based on the graph, the minimum starting potential that could be applied to the bioelectrode was determined as −0.80 V. Therefore, −0.80 V was fixed for further enrichment of the electron-consuming biocathode. Bicarbonate was used as carbon source starting from 16.5 day. A stable current was observed after 23.5 day. Sulfate test was performed at 26 day to check whether the biocathode was dominated by sulfate-reducing bacteria and depended on the compound to perform anaerobic respiration (Jeremiasse et al., 2012; Croese et al., 2014). The results showed little or no significant effect of the sulfate when the concentration was reduced from 5 mM to zero. Therefore, the cathode potential was further reduced to −0.9 V and a remarkably current density dropped was noticed between 32 and 34 day. The current was resumed after 5 mM ${\text{SO}}_{4}^{2-}$ was reintroduced to the biocathode.

### Effects of cathodic potential on hydrogen production

**Figure 5:**
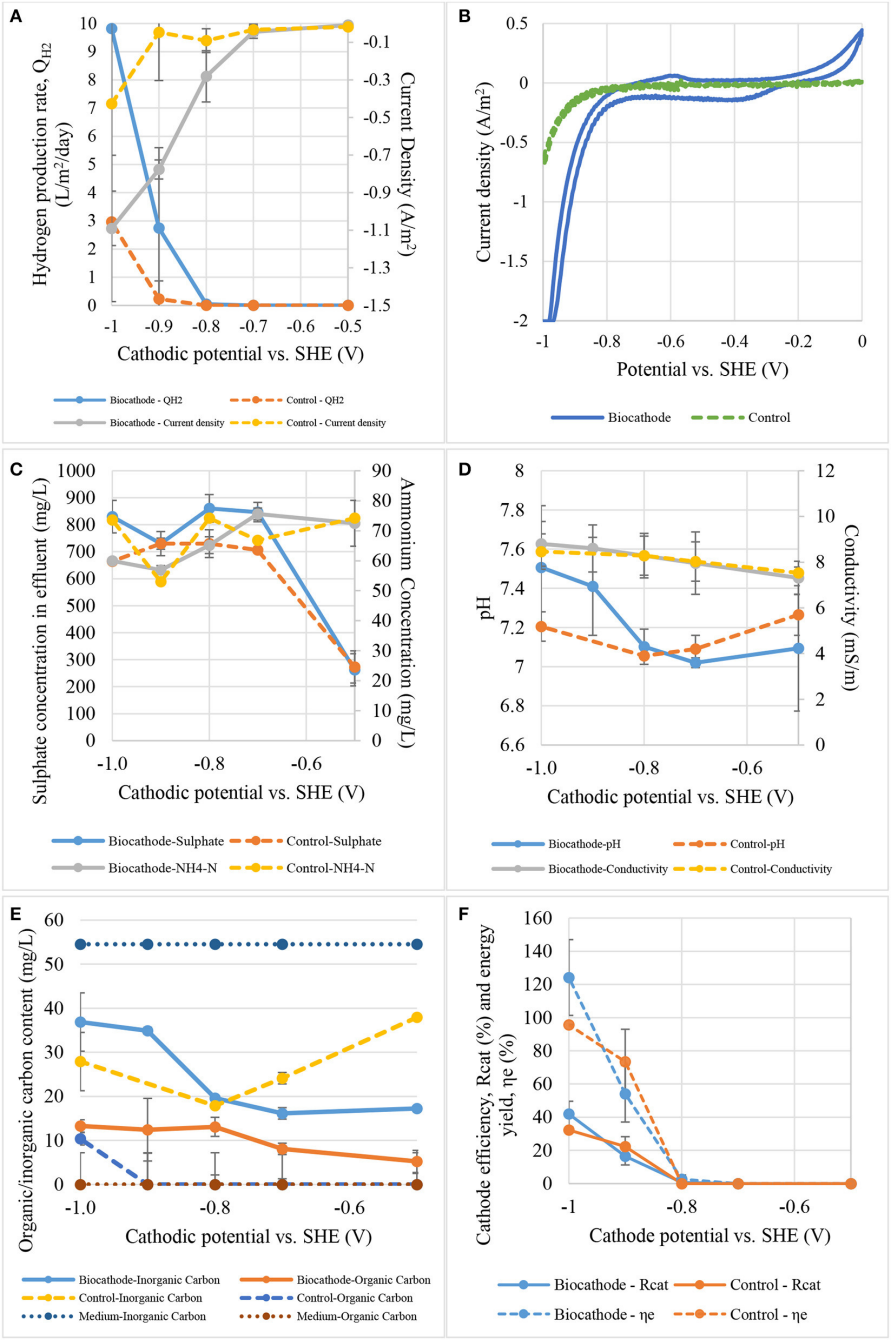
The effect of cathode potential on: **(A)** hydrogen production rate and current density, **(B)** catalytic activity, **(C)** sulfate and ammonium contents, **(D)** pH and conductivity, **(E)** total carbon content, and **(F)** cathode efficiency and energy yield.

The reactors were operated under different cathode conditions and performance between biocathode and abiotic cathode were compared. The cathode potentials was first fixed at −0.5 V before moving toward more negative potential until −1.0 V where a significant amount of gas was collected in the headspace. Each applied potential was fixed and applied for at least 2–3 days to obtain a stable current and hydrogen production. Figure 5A represents current density and hydrogen production rate from both control and biocathode. As shown in Figure 5A, biocathode hydrogen production was higher than control when the cathode potential was fixed at −0.8 V or below. No significant hydrogen production was observed in both biocathode and control when the potential was higher than −0.8 V. The biocathode produced almost 10 L/m^2^/day compared with the control cathode production of 3 L/m^2^/day at −1.0 V, evidencing biotic activity. The hydrogen production increased consistently with the external energy requirement for hydrogen evolution at lower potentials. The current density achieved was −1.10 A/m^2^ for biocathode compared to −0.45 A/m^2^ for the control, at a cathode potential of −1.0 V. Even though the reduction potential for hydrogen evolution from proton at standard condition is −0.41 V, the real operational reduction potentials are much more lower than the theoretical value (Lim et al., 2017). Potentials as low as −0.7 V and below were used to produce hydrogen as a result of overcoming overpotentials during the electron transfer to microbes (Rozendal et al., 2008; Jeremiasse et al., 2012; Jourdin et al., 2015). In additional, some studies applied even lower potentials than −0.7 V due to the different designs and configurations that possibly increased the overpotentials (Aulenta et al., 2012; Batlle-Vilanova et al., 2014; Liang et al., 2014; Luo et al., 2014; Lim et al., 2017).

Figure 5B shows the catalytic activity between biocathode and control (without inoculum) under the potential range of 0 to −1.0 V. Significant reduction activity was observed from −0.8 V and below. A small oxidation peak at −0.6 V was noticed when the voltammetry was scanned from −1.0 to 0 V. The peak was asserted as hydrogen oxidation reaction where the generated hydrogen (near −1.0 V) was re-oxidized under the outer membrane enzymes called hydrogenases (Aulenta et al., 2012). Furthermore, a small reduction curve at −0.3 V was also noticed and proved to be related to the process of inorganic to organic carbon conversion. Similar reduction peak was found in other CO_2_ conversion studies especially those for acetate production at the range between −0.3 and −0.6 V (Marshall et al., 2012; Blanchet et al., 2015; Patil et al., 2015; Bajracharya et al., 2017; Wenzel et al., 2018). Meanwhile, the control only showed reduction activity at −0.8 V and below and the activity was significantly lower than the biocathode. The catalytic properties proved biocathode growth on the electrode surface (Aulenta et al., 2012; Jourdin et al., 2015). Data suggests that hydrogen production was significant after cathodic potentials more negative than −0.8 V.

Figure 5C shows the variation in sulfate and ammonium content at different applied potentials. Lower potential was not necessary to increase the sulfate removal rate as fresh medium was continuously fed into the chamber (Jeremiasse et al., 2012; Luo et al., 2014). However, ammonium removal slightly increased at potentials lower than −0.8 V as ammonium acted as nitrogen source for microbial cell construction which could be much more important than sulfate as electron terminal acceptor.

Ph and conductivity are simpler indicators to biocathode activities. Figure 5D presents the pH and conductivity according to cathodic applied potentials. The pH of catholyte in biocathode remained at 7.0 between −0.5 and −0.8 V but start to increase to 7.5 when the potential was further decreased to −1.0 V depending on hydrogen evolution. The rate of pH increases was disproportional to the applied potential. However, catholyte pH in control fluctuated slightly between 7.0 and 7.3. Conductivity for both biocathode and control was increased vaguely from 8.0 to 9.0 mS/m when potential was dropped from −0.5 to −1.0 V. It is crucial to control the pH at neutral or slightly acidic to maintain the biocathode performance in producing hydrogen (Rozendal et al., 2008; Jeremiasse et al., 2010). This is because proton was continuously removed to produce hydrogen causing the increases of pH value.

Figure 5E shows inorganic and organic carbon contents of biocathode and control effluents. Bicarbonate as an inorganic carbon can be converted to acetate by homoacetogens to generate energy for growth (Bar-Even, 2013; Schuchmann and Muller, 2014; Mohanakrishna et al., 2015). Acetate was then could be used by SRB as the carbon source (Aulenta et al., 2012; Jeremiasse et al., 2012). This means that bicarbonate was converted to cell materials of homoacetogens and SRB, and to acetate. As observed from the Figure 5E, inorganic carbon content went up faster than organic carbon when more negative potential was applied to biocathode. It might due to external energy supply shifted the metabolic pathways from acetogenic energy conversion to direct electron uptake from high potential cathode or because of the excessive external energy at lower potential was more favored in SRB compared to acetate (Venzlaff et al., 2013). As a results, inorganic carbon was not in used causing the accumulation of inorganic carbon at lower applied potentials. However, cell yield is usually low in this system and the conversion to cell materials can be ignored. There was a 20–45% increase compared to fresh medium indicated a formation of organic carbon generated in the biocathode (data not shown). Interestingly organic carbon content from biocathode was higher compared to control with the same applied potential. While the potentials were low, the differential of the content was significant but start to converge when reaching −1.0 V which showing the shift of CO_2_ to electron uptake dependent and favored the SRB instead of acetogens. However, there was no consistent pattern in inorganic carbon removal in controls. Standard reduction potential for hydrogen evolution at neutral pH is −0.41 V while acetate is higher around −0.28 V (Geelhoed et al., 2010; Rabaey and Rozendal, 2010; Lim et al., 2017). Due to thermodynamic considerations, hydrogen-producing biocathode not only produce hydrogen but they could promote acetate production as well. In our experiments, more negative potentials were used starting from −0.5 to −1.0 V and not only inducing abiotic reduction of bicarbonate to organic carbon but also hydrogen evolution. Nevertheless, the reduction potentials favored the biocathode compared to control because the rate of hydrogen production and current density were much higher in biocathode.

Figure 5F shows cathode efficiency and energy yield of biocathode under different applied potentials. The values of cathodic efficiency between control and biocathode were almost similar within the tested applied potentials. Nevertheless, significant difference was only observed below −0.8 V rising from 0 to about 40% at −1.0 V. The energy yield also showed the same trend as cathodic efficiency with dramatically rise below −0.8 V. However, the energy yield for biocathode (120%) was slightly higher than control (100%) at −1.0 V. The value of energy yield was more than 100% as the calculation taking account of external power rather than both anode and external power contributions (Lim et al., 2017). Besides, the experiments were focused on cathode reaction which were conducted in half-cell setup instead of whole cell causing inaccuracy in the calculation. Even though the values were overestimated, they provided quantitative comparisons between the control and biocathode.

### Effects of sulfate concentration on hydrogen production

**Figure 6:**
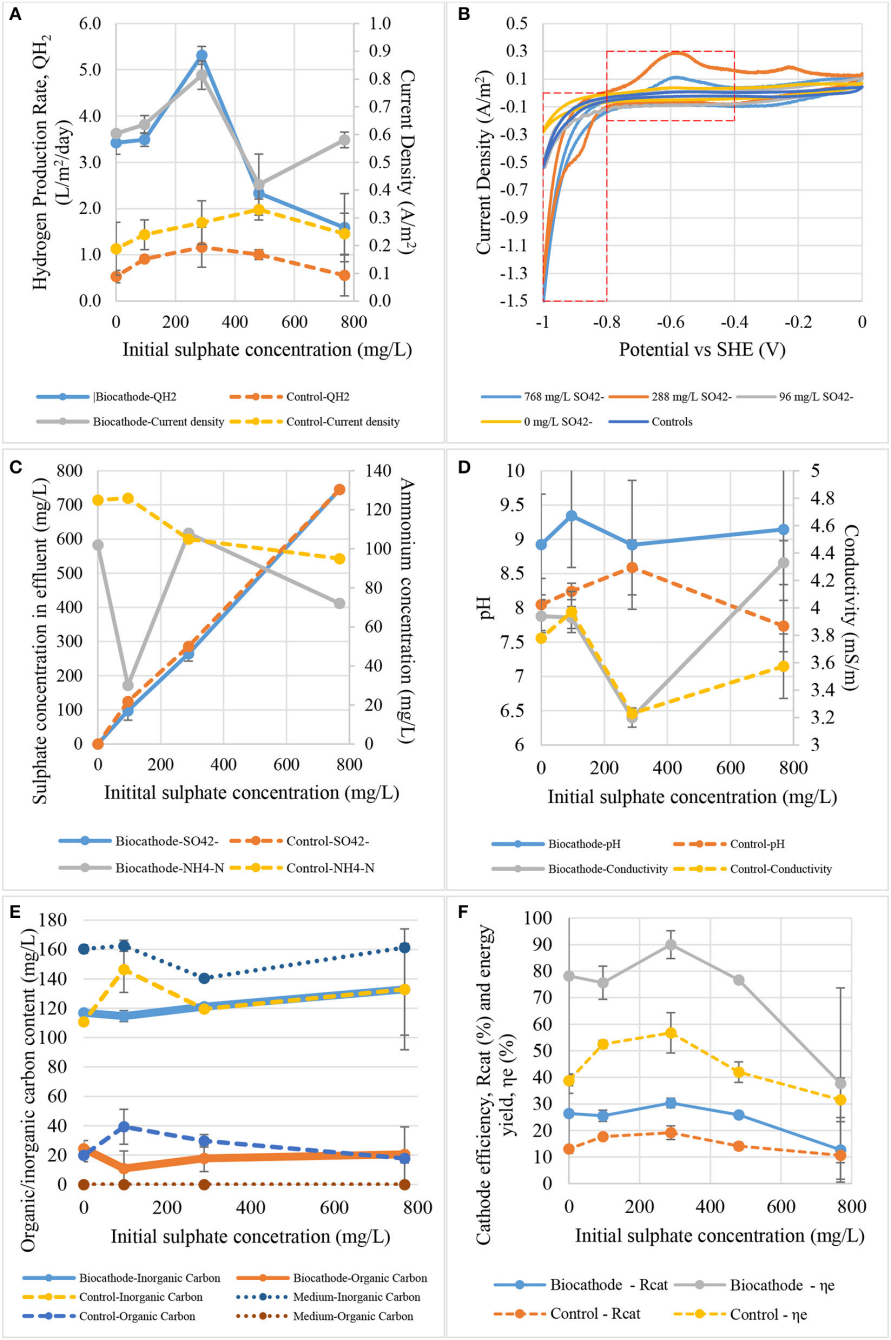
The effect of initial sulfate concentration on: **(A)** hydrogen production rate and current density, **(B)** catalytic activity, **(C)** sulfate and ammonium contents, **(D)** pH and conductivity, **(E)** total carbon content, and **(F)** cathode efficiency and energy yield.

Figure 6A shows the effect of sulfate concentration to current density and hydrogen production rate at cathodic potential of −1.0 V. In the test, both peak hydrogen production rate and current density occurred at a sulfate concentration of 288 mg ${\text{SO}}_{4}^{2-}$/L. The peak hydrogen production rate and current density were 5.3 L/m^2^/day and −0.81 A/m^2^ respectively. Meanwhile the control remained almost stagnant throughout this test. Hydrogen production rate could be highly depended on the sulfate concentration due to fact that the sulfate might favor certain microorganisms like SRB. It is commonly known that high substrate concentration could limit or saturate metabolic reactions in living cells. The sulfate reduction in this case was limited by low sulfate concertation (<288 mg ${\text{SO}}_{4}^{2-}$/L). The effect of sulfate inhibition began to observe after 288 mg ${\text{SO}}_{4}^{2-}$/L where the current density and hydrogen production rate started to plummet. At this stage, SRB would reduce sulfate preferentially over proton under unlimited bicarbonate source. Extra reducing power or lower cathodic potential was needed to support the reduction of sulfate. Therefore, the hydrogen production was disproportional to the sulfate concentration as more electrons are used to reduce sulfate rather than protons at high sulfate concentration. The present of sulfate is important for SRB to outcompete other anaerobes, including methanogens and fermentative bacteria in the anaerobic environments (Muyzer and Stams, 2008; Madigan et al., 2014). When the sulfate is quantitatively low, methanogens could dominant in the community. However, SRB could survive at very low amount of acetate as carbon source compared to the methanogens, and therefore, they will coexist with homoacetogens when acetate is not available (Singleton, 1993; Muyzer and Stams, 2008).

Figure 6B shows the cyclic voltammograms of the biocathode on the sulfate concentration. Based on the results, we believe sulfate could be considered as one of the key parameters in this study. It can be seen from the figure that the evolvement of specific catalytic peaks at −0.6 and −1.0 V was actually affected by the sulfate concentration. Both peaks were postulated catalyzing hydrogen oxidation and hydrogen evolution related to the species of sulfate-reducing bacteria (Aulenta et al., 2012; Lim et al., 2017). Moreover, significant hydrogen oxidation and reduction peaks were observed at 288 mg ${\text{SO}}_{4}^{2-}$/L. The oxidation peak was believed to be related to reversible electrochemically active periplasm enzymes or proteins called hydrogenases. Hydrogenase can be found in many microorganisms included SRB, acetogens and methanogens and catalyze hydrogen production and/or utilization. The higher oxidation peak at 288 mg ${\text{SO}}_{4}^{2-}$/L was due to the increased hydrogenase content on the biocathode. In the cyclic voltammogram, the hydrogenases performed instant hydrogen oxidation around −0.6 V which was generated at −1.0 V when the applied potential moved from −1.0 to 0 V. The increases in hydrogenase activity was also supported by the evidence that the maximum hydrogen production rate was at the same sulfate concentration. Even though hydrogen catalysis (by comparing the CV tails at −1.0 V) was slightly higher at 768 compared to 288 mg ${\text{SO}}_{4}^{2-}$/L, the hydrogen oxidation peak at −0.6 V was not as high as at 288 mg ${\text{SO}}_{4}^{2-}$/L. This could be due to the substrate inhibition on the hydrogenases (Aulenta et al., 2012; Batlle-Vilanova et al., 2014). It was believed that this enzyme posed an on set potential at least at −0.6 V while an extra −0.4 V (standard reduction potential for hydrogen evolution) should be invested to produce hydrogen (Lim et al., 2017).

Figure 6C exhibits sulfate and ammonium concentration in the effluent depend on initial sulfate concentrations. Sulfate concentration as low as 96 mg/L was actually good for ammonium removal. It means that sulfate and ammonium should be presented in the same time but not in high concentrations for a better biocathode reactions. Ammonium was depleting faster at 96 mg ${\text{SO}}_{4}^{2-}$/L that the other concentrations and became a limiting factor to block the current and hydrogen production as shown in Figure 6A. Surprisingly, the current and hydrogen production rate reached a peak at 288 mg ${\text{SO}}_{4}^{2-}$/L but decrease after higher sulfate concentration. Substrate inhibition could be the main factor restricting the activities and not necessary for better hydrogen production as long as the sulfate was presented in the environments (Jeremiasse et al., 2012).

Ph and conductivity values were plotted relatively to sulfate concentration in Figure 6D. The pH increased in biocathode explains protons were utilized and removed from the catholyte to produce hydrogen. The biocathode pH fluctuated between 8.9 and 9.3 which was higher than initial medium pH around 7.0. However, the control pH was slightly lower than the biocathode pH with the value in between 7.7 and 8.6. The higher the pH values indicated that more protons were removed during reduction process and biocathode activity. At this point the pH values were increased remarkably from neutral to about 9.0. This means the added 50 mM phosphate buffer (PBS) in the medium wasn't the best option for controlling but managed to prevent a dramatically changes of pH. LaBelle et al. (2014) lowered catholyte pH to around 5.0 in order to increase hydrogen production in acetogen and SRB dominated mixed community. Acetogen domination in biocathode could be a problem as they ceased the production of hydrogen. Therefore, *Acetobacterium* dominated biocathode was controlled at certain level in repeatedly exposure to acidic condition to increase hydrogen production rate (LaBelle et al., 2014; LaBelle and May, 2017). Meanwhile, lower pH could also mean to provide more proton for hydrogen and acetate production. Surprisingly, conductivity values followed the trend of hydrogen production and current density. This is different from the effect of applied potentials where the conductivity and pH values did not change dramatically.

Figure 6E shows the inorganic/organic content relatively to sulfate concentration. The organic carbon content in control and biocathode effluent was remained almost the same without any significant different when the sulfate concentration was increased. The main purpose of this results was to notice any relevant connection between bicarbonate and sulfate roles in the biocathode. From the results, there was no clear connection between the tested parameter. Either bicarbonate or sulfate was required by two different community and no competitions was exist between them for sulfate and bicarbonate in the same time. The evidence concretes the idea that bicarbonate was necessary for some autotroph community in biocathode to produce organic carbons (Mohanakrishna et al., 2015). The organic carbons were then utilized by SRB to produce hydrogen with external reducing power for cathode (Jeremiasse et al., 2012; Zaybak et al., 2013).

Figure 6F shows the effects of sulphate concentration to hydrogen recovery efficiency and energy yield for biocathode and control. Overall, the efficiency and yield values of biocathode were higher than control and peaked at 288 mg ${\text{SO}}_{4}^{2-}$/L. The biocathode efficiency and energy yield were calculated as 30 and 90%, which are higher compared to the control that only achieved up to 20 and 56% at the peak. The biocathode energy yield dropped faster than the control might be due to the lower hydrogen production when large portion of supplied energy was utilised by the biocathode to reduce sulphate instead of proton. In contrast, low sulphate concentration (<288 mg ${\text{SO}}_{4}^{2-}$/L) limited the hydrogen production indicated sulphate is one of the important reactants or compounds for the biocathode in the proton reduction reaction.

### Effects of bicarbonate content on hydrogen production

**Figure 7:**
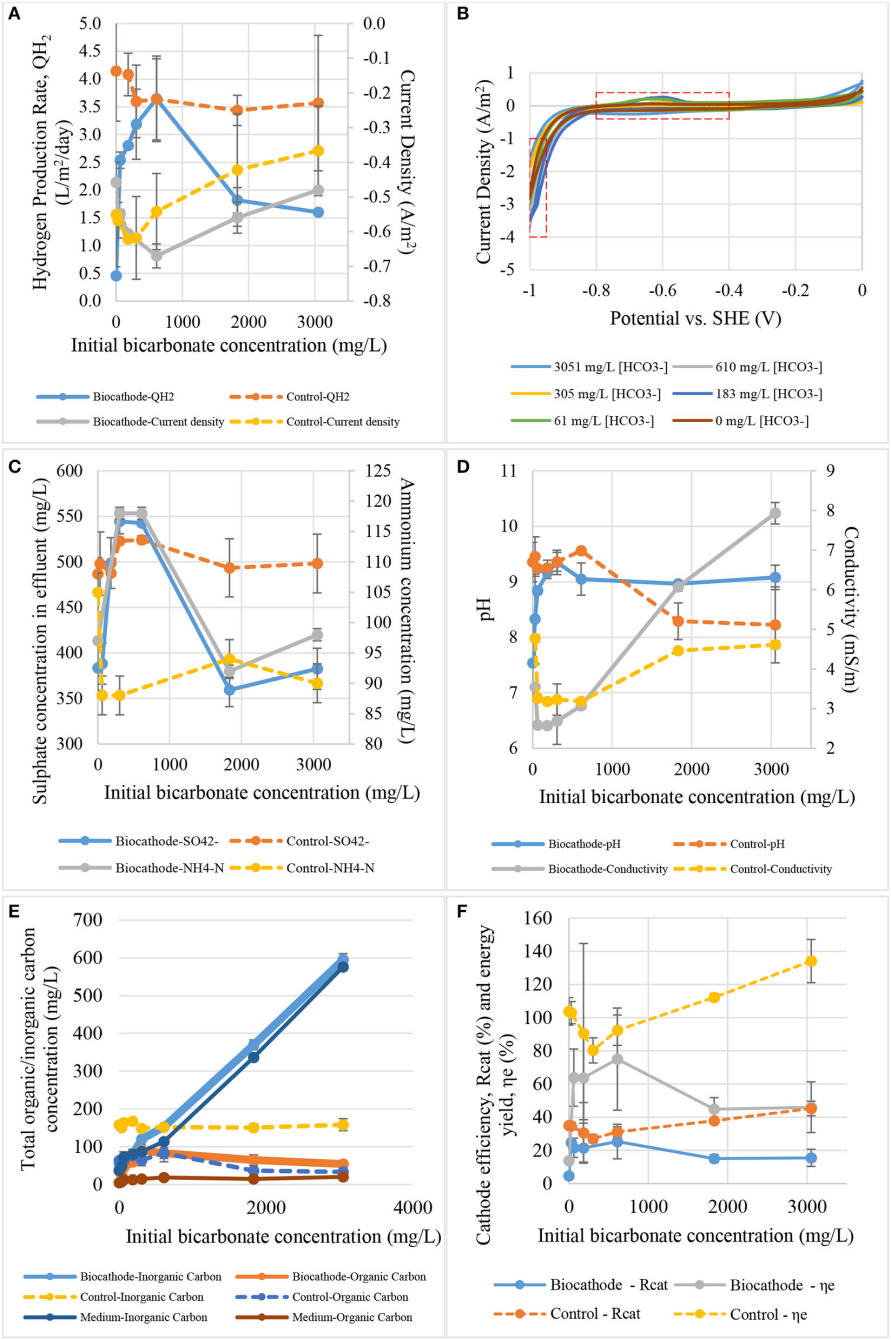
The effect of initial bicarbonate concentration on: **(A)** hydrogen production rate and current density, **(B)** catalytic activity, **(C)** sulfate and ammonium contents, **(D)** pH and conductivity, **(E)** total carbon content, and **(F)** cathode efficiency and energy yield.

The effect of bicarbonate concentration to current density and hydrogen production rate is shown in Figure 7A. The bicarbonate test showed that a concentration of 610 mg ${\text{HCO}}_{3}^{-}$/L gave the maximum hydrogen production rate of 3.6 L/m^2^/day and the maximum current density of −0.67 A/m^2^. The control hydrogen production rate in this test was almost the same after 305 mg ${\text{HCO}}_{3}^{-}$/L. One of the speculation is that there is no biofilm was growth or attached on the surface of control cathode. Hence, the transportation of protons from bulk solution to control cathode surface was faster than in biocathode. Abiotic hydrogen production rate was remained stagnant at 3.6 L/m^2^/day after 305 mg ${\text{HCO}}_{3}^{-}$/L. Meanwhile, hydrogen production in biocathode peaked at 305 mg ${\text{HCO}}_{3}^{-}$/L with the production rate equal to 3.6 L/m^2^/day.

Figure 7B shows the cyclic voltammograms of the biocathode in different bicarbonate concentrations. Low bicarbonate concentration (61 and 183 mg ${\text{HCO}}_{3}^{-}$/L) was actually good for biocathode catalytic activity as they induced the highest hydrogen oxidation peak. However, only 610 mg/L ${\text{HCO}}_{3}^{-}$/L promoted the highest hydrogen production rate and current density as shown in Figure 7A. If the interaction of microbial community in the biocathode was true, acetogens that produced short-chain fatty acids for the hydrogen producing bacteria could be saturated with the inorganic carbon concentration at 610 mg ${\text{HCO}}_{3}^{-}$/L or higher (Su et al., 2013; LaBelle and May, 2017). Maximum fatty acid was converted at this concentration. Thus, the hydrogen production rate and current density were the highest at this bicarbonate concentration. Higher catalytic activity at −0.6 V did not necessary means it could promote high hydrogen evolution and the interaction of biocathode microbes should be taking into consideration.

Figure 7C illustrates the profile of effluent sulfate and ammonium concentration to initial sulfate concentration. Bicarbonate worked as carbon source is crucial to support biocathode growth. The quantity could affect sulfate and ammonium removal especially at 610 mg ${\text{HCO}}_{3}^{-}$/L. The value is the optimum concentration because it gave the maximum current and hydrogen production. As we could see in Figure 7C the sulfate removal in biocathode was gone up at low bicarbonate concentration but decreased after reaching the peak. It was revealed that either the fixed sulfate concentration was not sufficient to support the rate of biocathode activities when bicarbonate concentration was high. More sulfate was required for the reactions.

The effect of bicarbonate to pH and conductivity value is presented in Figure 7D. Carbonate species could act as buffer system to maintain the pH as observed in control. The pH was maintained after 1,831 mg ${\text{HCO}}_{3}^{-}$/L. For the biocathode, bacterial growth in biofilm usually is much lower than free-living bacteria and cell yield is low in anaerobic bacteria. These mean that the effects of carbonate might not be related to the bacterial growth. Therefore, the hydrogen production rate between control and biocathode was not significantly different between each other. The only comparable performance was the current density where the biocathode required lower energy that the control. About 0.15 A/m^2^ different between both control and biocathode after 305 mg ${\text{HCO}}_{3}^{-}$/L. Second explanation is that at least two biotic steps was need to produce hydrogen. As we known that SRB which responsible for the hydrogen production are chemoorganotrophs and could not use inorganic carbon to growth (Muyzer and Stams, 2008). Therefore, autotrophic acetogens become important to in the community to produce acetate from bicarbonate which in turn consumed by SRB. Some literature also suggested that the hydrogen and acetate production were coexistent in hydrogen-producing biocathode (Su et al., 2013; LaBelle et al., 2014; LaBelle and May, 2017). In additional to the PBS, Liang et al. (2014) suggested that bicarbonate could also enhances electric migration of proton when more H^+^ was release from ${\text{HCO}}_{3}^{-}$ and accelerated hydrogen evolution. This explained why the conductivity was getting lower at peak hydrogen production rate. Bicarbonate may contribute to the conductivity values. Catalytic activity of hydrogen production could actually utilized the proton and CO_2_ derived from ${\text{HCO}}_{3}^{-}$, driving the conductivity value low as ${\text{HCO}}_{3}^{-}$ was consumed.

Figure 7E shows the inorganic/organic carbon conversion from different bicarbonate concentration. Bicarbonate concentration was increased constantly to monitor the effect on the biocathode. Organic carbon concentration increased until it reached a peak at 305 mg ${\text{HCO}}_{3}^{-}$/L. The bicarbonate was essential in this study as a carbon source for microbial growth (Luo et al., 2014; Jourdin et al., 2015; Mohanakrishna et al., 2016). Hydrogen production also reached a maximum point at this concentration. This postulated that possibly of carbonates consumed by autotrophs such as acetogens to produce organic carbons which in turn used by SRB to produce hydrogen. Once the bicarbonate concentration excess 305 mg ${\text{HCO}}_{3}^{-}$/L, the hydrogen production rate dropped dramatically as shown in Figure 7A. Substrate inhibition may occurred within the biofilm when acetogens produce excessive organics carbons and decrease hydrogen production in SRB (Croese et al., 2014; Bajracharya et al., 2017; LaBelle and May, 2017). On one hand, organic carbons content and removal in biocathode seems to peak at 305 mg ${\text{HCO}}_{3}^{-}$/L which is proportional to hydrogen production rate. On the other hand, the organic carbon content and removal in control were remarkably lower compared to the biocathode. The trend of changing was negligible and lightly shifted relative to the bicarbonate concentrations.

Figure 7F presents the effects of bicarbonate concentration to hydrogen recovery efficiency and energy yield for biocathode and control. Surprisingly, both efficiency and yield values for biocathode were lower than control. Higher bicarbonate concentration did not assist the biocathode in hydrogen production. Instead, the efficiency and yield decreased after the bicarbonate concentration was more than 610 mg ${\text{HCO}}_{3}^{-}$/L. This is because higher bicarbonate concentration could inhibit the biocathode reaction activities as discussed in the paragraphs above. Meanwhile, the efficiency and yield values increased proportional to bicarbonate concentration in abiotic control probably of the buffering properties of bicarbonate (Liang et al., 2014).

### Bottlenecks and beneficial applications of hydrogen-producing biocathode

It is believed that microbial community in hydrogen-producing biocathode should contain key enzyme, hydrogenases in order to catalyst hydrogen evolution from protons (Geelhoed et al., 2010; Croese et al., 2011; Rosenbaum et al., 2011; Jourdin et al., 2015; Kim et al., 2015). Sulfate-reducing bacteria (SRB) belong to *Desulfovibrio* sp. was then found abundant in the biocathode which contain active hydrogenase enzymes in its cytoplasm and periplasm (Croese et al., 2014). According to the conventional information, SRB poses energy conservation mechanism called hydrogen cycling mechanism in sulfate reduction (Kim and Gadd, 2008; Madigan et al., 2014). The mechanism happens in anaerobic condition by oxidizing organic compounds like lactate and ethanol as electron donors for sulfate reduction. However, there was no organic matter only inorganic carbon like carbonates introduced to hydrogen-producing biocathode. To replace the organic matter, external energy source was required to provide the reducing power to the biocathode. In our study, it was found that at least −0.8 V vs. SHE was required to make the biocathode feasible for hydrogen evolution (Figure 5A). The potentials provided sufficient exergonic energy to overcome overpotentials in the system and to facilitate electron transfer from electrode to electrochemically-active microbes. These microbes normally contain membrane-bound complexes such as cytochrome C, Fe-S protein, oxidoreductase and periplasm enzymes that could receive the electrons (Choi and Sang, 2016). As a result, the microbes could perform the metabolic process and initialize the electron transport-chain reactions and generate hydrogen included trace amount of organic carbon.

From thermodynamic point of view, standard reduction potential, *E*°′ for hydrogen evolution from proton, H^+^/H_2_ is −0.41 V at neutral pH. In real case scenario, potentials lower than this value were normally applied to biocathode to overcome overpotential and activation loss (Rozendal et al., 2008; Aulenta et al., 2012; Batlle-Vilanova et al., 2014; Jourdin et al., 2015; Lim et al., 2017). In additional to the proton reduction under energy conserving hydrogenases in *Desulfovibrio* sp. respiration, sulfate is also an important element as final terminal electron acceptor. The *E*°′ of ${\text{SO}}_{4}^{2-}$/H_2_S is −0.35 V which the potential is slightly higher than reduction of protons to hydrogen [*E*°′ ${\text{SO}}_{4}^{2-}$/H_2_S −0.35 V is calculated based on *E*°′ ${\text{SO}}_{4}^{2-}$/${\text{HSO}}_{3}^{-}$ −0.52 V and *E*°′ ${\text{SO}}_{3}^{2-}$/H_2_S −0.17 V] (Madigan et al., 2014). Sulfate reduction will be dominated in the present of high sulfate concertation as less energy is required and causing less hydrogen evolution. Even in real environmental concentration is considered, the couple of H^+^/H_2_ is still more negative than ${\text{SO}}_{4}^{2-}$/HS^−^ (*E*°′ of H^+^/H_2_ is −0.27 V at 1 Pa of H_2_ and ${\text{SO}}_{4}^{2-}$/HS^−^ is −0.20 V at 0.1 mM HS^−^; Keller and Wall, 2011). In recent development, it has been proven that the potentials required of bioelectrochemically hydrogen evolution is lower than sulfate reduction (Luo et al., 2014; Zheng et al., 2014). Under fed-batch mode, the cathode potentials for sulfate reduction ranged between −0.6 to −1.0 V (Luo et al., 2014). Meanwhile, significant hydrogen evolution potentials were around −0.8 to −1.2 V (Aulenta et al., 2012; Batlle-Vilanova et al., 2014; Lim et al., 2017). Slightly more positive potential around −0.7 V were also used to generate hydrogen from biocathode but under a feed-controlled system in the anode and cathode. The purpose of the system is to eliminate mass transport limitation and overpotential losses that occurred in a batch system (Rozendal et al., 2008; Jeremiasse et al., 2010).

In this study, it is interesting to show that bioelectrochemically hydrogen production was sulfate-dependent. The hydrogen production rate was recorded by varying the cathode potentials, sulfate and bicarbonate concentrations as shown in this study. In spite of that, operational potentials have been well studied in hydrogen-producing biocathode and are predictable using the thermodynamic information (Geelhoed et al., 2010; Keller and Wall, 2011; Jafary et al., 2015; Choi and Sang, 2016). In addition to the potential, carbonate concentration might not literally affected by the BES performance in this study. This is because of anaerobic bacteria normally grow slowly on biocathode compared to free-living bacteria or in aerobic condition (Kim and Gadd, 2008; Madigan et al., 2014). SRB are chemolithotrophic bacteria that required organic matters like acetate to growth. Some studies reported the requirement of organic matter in hydrogen-producing biocathode by adding acetate in carbonate-containing medium (Liu et al., 2005; Jeremiasse et al., 2012; LaBelle et al., 2014). It is suspected that this bacteria actually live syntrophically with acetogens which are autotrophs. The growth of these autotrophs were even lower if they involved in the biocathode activities such as acetogens and the accumulation of biomass would be redundant (Su et al., 2013; Mand et al., 2014). Jeremiasse et al. (2012) tried to test the acetate and sulfate effects on hydrogen-producing biocathode by feeding the medium with and without acetate or sulfate. It is interesting to point out that the current density supplied to the system was slightly lower at the beginning for sulfate-fed biocathode but overtook the control biocathode after 20 days (Jeremiasse et al., 2012). Based on this reason, it is believed that electron bifurcation couple process occurred from both protons and sulfate reduction simultaneously. Electron bifurcation has been emerged and recognized as the third important biological energy conservation mechanism in the last decades after the two fundamental mechanisms, substrate level phosphorylation and electron transport-linked phosphorylation were unable to explain thermodynamically unfavorable reactions (Buckel and Thauer, 2013; Peters et al., 2016).

**Figure 8:**
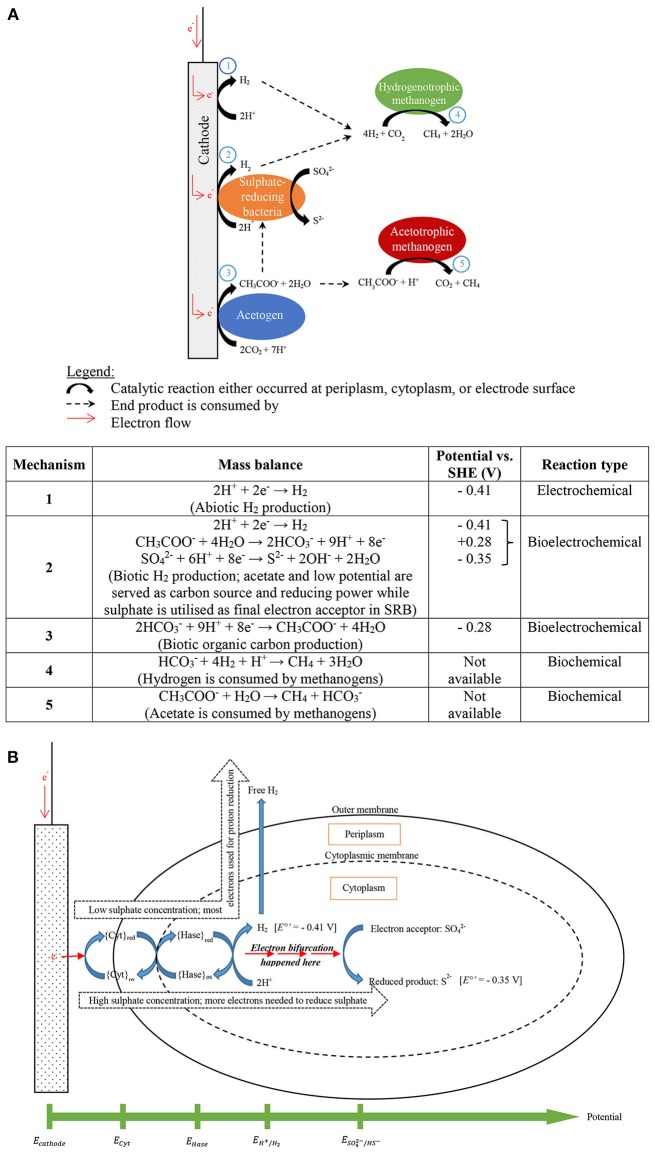
**(A)** Proposed electron flow and possible final destinations of the supplied electrons being utilized in producing various end products (modified after Mand et al., 2014), **(B)** description of electron bifurcation flow in sulfate-reducing bacteria-dominated biocathode to generate hydrogen and reduce sulfate.

In the review, Keller and Wall (2011) claimed that *Desulfovibrio* sp. produce hydrogen during sulfate reduction with ethanol. This involves electron bifurcation and *Desulfovibrio* sp. have energy conserving hydrogenases As *Desulfovibrio* sp. oxidize ethanol reducing NAD^+^ to NADH (*E*°′ = −0.320 V), NADH is bifurcated to reduce sulfate and proton (Ramos et al., 2015). In the paper, Ramos et al. (2015) found hdrCBA-flxDCBA gene cluster is presented in many different phyla including electrochemically active microbes, *Desulfovibrio* sp. and *Geobacter* sp. This gene is responsible for transcribing flavin oxidoreductase (FlxABCD) and heterodisulfide reductase (HdrABC) to perform flavin-based electron bifurcation (FBEB). Both enzymes are involved in producing reducing carriers for hydrogen evolution and sulfate reduction. Proton reduction to hydrogen is catalyzed by energy-conserving hydrogenase with the reducing carriers. It is hypothesized that at low cathode potential sulfate is reduced without hydrogen production, and if hydrogen is produced it is not sulfate-dependent. When the cathode potential was not low enough to reduce proton, electrons were bifurcated reducing both high and low redox potential electron carriers. The former is used to reduce sulfate and the latter to reduce proton conserving in both reduction reactions. Based on these facts, it is believed that hydrogen production would be inhibited in the presence of sulfate or sulfate-dependent because SRB conserve more energy reducing sulfate than reducing proton as shown in Figure 6. Figure 8 describes the possible electron bifurcation flow for SRB growth on cathode used to reduce proton and sulfate. Lower sulfate concentration is actually good for SRB respiration (<288 mg/L) and promoted proton reduction. The hydrogen evolution decrease dramatically when more sulfate was added (>288 mg/L) as more electrons were utilized by reducing sulfate instead of protons.

Last but not least, the finding of the sulfate-dependent hydrogen-producing biocathode has raised the question; what type of wastewaters can be treated by using this technology? The sulfate dependency was due to the SRB domination in the biocathode and a specific range of sulfate concentration was required to maintain the balance and functionality of the biocathode to produce hydrogen while reducing sulfate. Domestic wastewater usually contain low amount of sulfate between 20 and 60 mg/L, although the concentration can be up to 500 mg/L for industrial wastewater (Lens et al., 1998; Moussa et al., 2006). Conventional sulfate removal technology benefits from the presence of SRB to treat domestic and industrial wastewaters. The benefits include reducing sludge accumulation and pathogen content (if present), removing heavy metals and as anaerobic digestion pre-treatment (van den Brand et al., 2015). In the present study, an “optimum” sulfate concentration was 288 mg/L which generated the maximum hydrogen volume. It is recommended to use domestic wastewater to enrich and maintain a hydrogen-producing biocathode, because low amounts of organic compounds and sulfate make it a better medium to enhance the growth of SRB. (Jeremiasse et al., 2012; Lee et al., 2014).

### Drawbacks on low potential, mass transport limitation and long term operation

**Figure 9:**
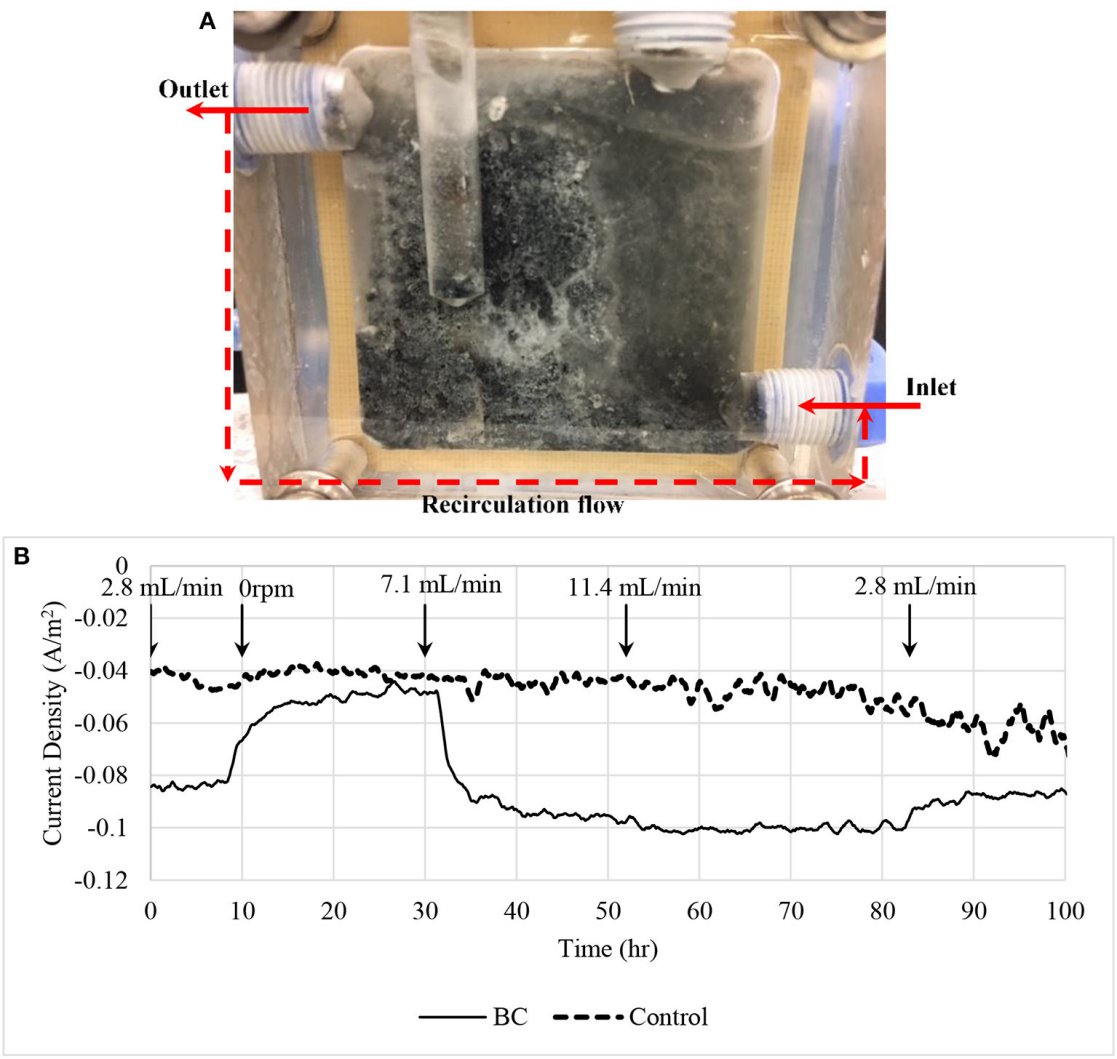
**(A)** Biocathode after the experiments. White crystallization and black biomass were appeared on the surface of the electrode causing current density dropped. **(B)** Current density affected by mass transport limitation. A recirculation flow line was connected between inlet and outlet to recycle the catholyte in order to reduce the mass transfer limitation. A control using 7.1 mL/min recycle flow rate was included in the figure for comparison purpose.

At the end of experiments, white precipitations could be observed from cathodic chamber (Figure 9A). The precipitated compounds were attached along with biomass on the surface of cathode and caused the biocathode performance drop over time. We believe the precipitations that crystallized on the cathode surface was a form of alkali phosphates due to low reduction potentials (Jeremiasse et al., 2010). Moreover, recycle flow was connected between outlet and inlet in order to reduce mass transport limitation between bulk solution and the biocathode. Figure 9B shows the relationship between current density and the flow rate. Four flow rates were used to test the mass transport limitation: 0, 2.8, 7.1, and 11.4 mL/min. When zero flow rate was applied to the chamber, the current density reduced significantly. Flow rate 7.1 mL/min was selected to use in the experiments as it generated almost similar current density compared to the higher flow rate 11.4 mL/min.

**Figure 10:**
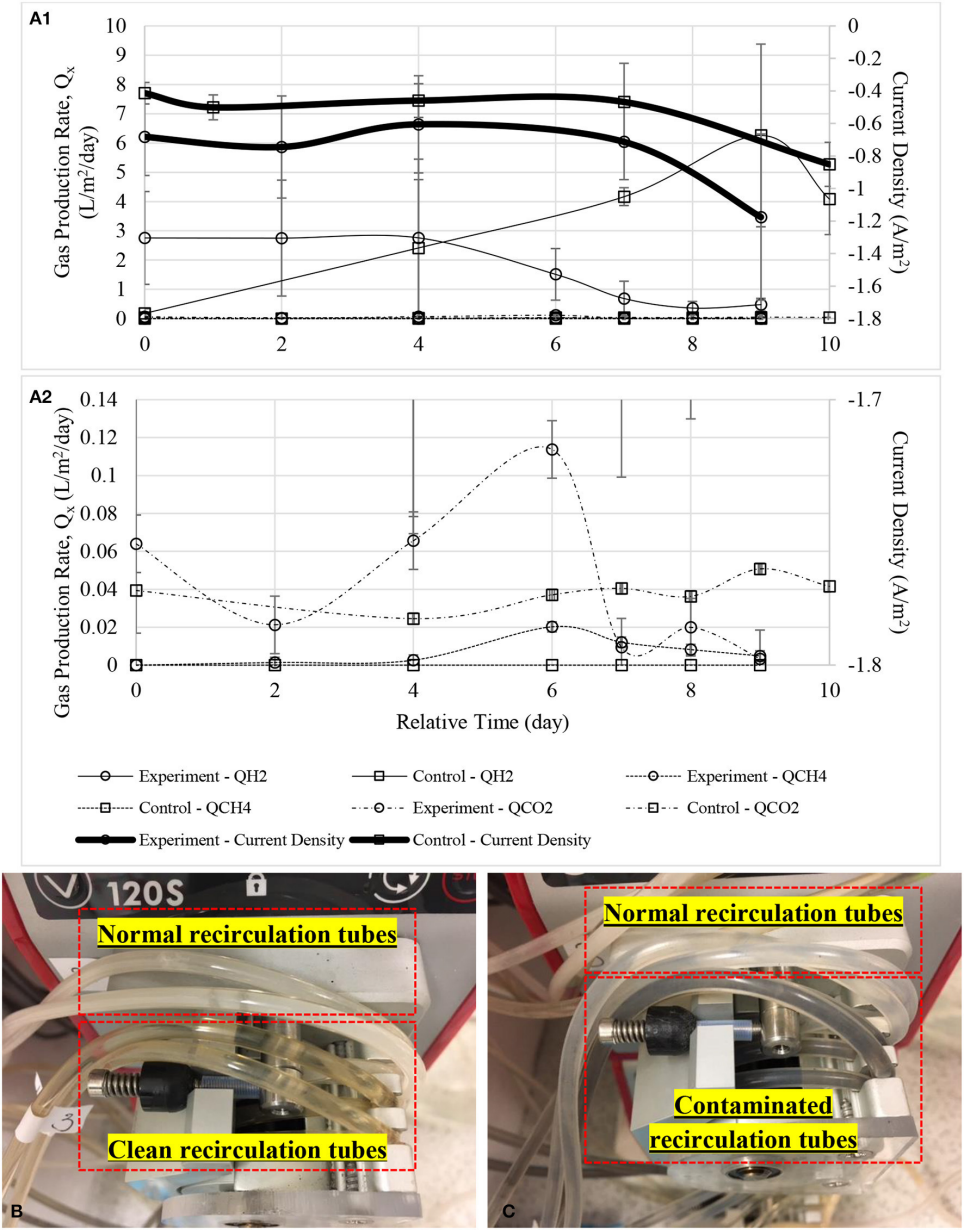
**(A1)** Gas production rate of the defected MECs with a zoom-in figure **(A2)**. Noted that hydrogen production dropped dramatically even when CH_4_ was first detected at a very low concentration at day 6. Small amount of bicarbonate was probably released as CO_2_ or consumed by methanogens. Timeline was adjusted to zero for comparison purpose. **(B)** Upper tubes show white biofilm grew on the inner surface of the tubes while lower tubes were after cleaned and soaked with disinfectant, Virkon, and **(C)** upper tubes were the normal biocathode recirculation tubes while black color biofilm was observed in the lower tubes when methane started to detect in gas samples.

The risk of enriched biocathode contaminated by methanogens under a hydrogen-rich environment after a long time operation have been previously reported (Wagner et al., 2009; Kyazze et al., 2010; van Eerten-Jansen et al., 2015; Bajracharya et al., 2017). Figure 10A shows performance dropped when methane was first detected in biocathode after 120 days of operation (further data not shown). Hydrogen production dropped remarkably after methane was detected in the biocathode at day 4. Current demand was also increased as more energy was required to support both hydrogen and methane production. Figure 10B shows the clean and normal recirculation tubes while Figure 10C shows the comparison between the normal and contaminated recirculation tubes when methane was first detected.

## Conclusion

This study revealed that applied potential, sulfate and inorganic carbon are vital parameters to promote hydrogen production in a biocathode of electrolysis cell. The optimum ratio of PBS: ${\text{HCO}}_{3}^{-}$: ${\text{NH}}_{4}^{+}$: ${\text{SO}}_{4}^{2-}$ in this study was determined as 950:610:90:288 mg/L (10:10:5:3 mM) for a biocathode sized 0.005 m^2^, operation volume 0.0025 m^3^ and applied potential −1.0 V vs. SHE in a continuous flow rate 0.1 mL/min. The information provided the first insight of how much carbon, nitrogen and sulfate sources that must be presented in the influent in order to provide better operational conditions. Even though the ratio may slightly vary according to the size of reactor, cell configuration and controlling system, the basic principle of how a biocathode catalyzes hydrogen under the influences of those main sources would still remain the same. Besides the ratio, external power supply was required to provide initial energy under low potential electrons to start the biocathode catalytic activity while sulfate served as final terminal electron acceptor to dispose the exhausted electrons. Inorganic carbon in the form of carbonates was added to the influent and worked as carbon backbone to support the growth of biocathode community. As organic carbon compounds were found in the biocathode effluents, it is believed that within the microbial community the inorganic carbon was consumed by acetogens to produce organic carbons such as acetate and then consumed by SRB as carbon source. Another significant finding is the present and quantity of sulfate did affect the hydrogen production in SRB-dominated biocathode. At high sulfate concentration, it could inhibit hydrogen production if the cathode potential was not low enough to reduce both sulfate and proton. The phenomena is similar to those electron bifurcation.

## Supplementary data statement

Data supporting this publication is openly available under an ‘Open Data Commons Open Database License’. (Lim et al., 2018) Additional metadata are available at: https://doi.org/10.17634/150659-2. Please contact Newcastle Research Data Service at rdm@ncl.ac.uk for access instructions.

## Author contributions

SL carried out the experiment and wrote the manuscript with support from EY and KS. BK conceived the original idea and suggested on the experiment framework. All authors provided critical feedback and helped shape the research, analysis and manuscript.

### Conflict of interest statement

The authors declare that the research was conducted in the absence of any commercial or financial relationships that could be construed as a potential conflict of interest.
